# Postoperative prolonged mechanical ventilation in patients with surgically treated pyogenic spondylodiscitis: a surrogate endpoint for early postoperative mortality

**DOI:** 10.1007/s10143-023-02016-1

**Published:** 2023-05-09

**Authors:** Jasmin E. Scorzin, Anna-Laura Potthoff, Felix Lehmann, Mohammed Banat, Valeri Borger, Patrick Schuss, Christian Bode, Hartmut Vatter, Matthias Schneider

**Affiliations:** 1 Department of Neurosurgery, University Hospital Bonn, Bonn, Germany; 2 Department of Anesthesiology and Intensive Care Medicine, University Hospital Bonn, Bonn, Germany; 3 Department of Neurosurgery, BG Klinikum Unfallkrankenhaus Berlin gGmbH, Berlin, Germany

**Keywords:** Prolonged mechanical ventilation, Pyogenic spondylodiscitis, Stabilization surgery, Mortality

## Abstract

Surgical procedures with spinal instrumentation constitute a prevalent and occasionally highly indicated treatment modality in patients with pyogenic spondylodiscitis (PSD). However, surgical therapy might be associated with the need of prolonged postoperative intensive care medicine which in turn might impair intended operative benefit. Therefore, we analyzed prolonged mechanical ventilation (PMV) as an indicator variable for such intensive care treatment with regard to potential correlations with mortality in this vulnerable patient cohort. Between 2012 and 2018, 177 consecutive patients received stabilization surgery for PSD at the authors’ neurosurgical department. PMV was defined as postoperative mechanical ventilation of more than 24 h. A multivariable analysis was performed to identify independent predictors for 30-day mortality. Twenty-three out of 177 patients (13%) with PSD suffered from postoperative PMV. Thirty-day mortality rate was 5%. Multivariable analysis identified “spinal empyema” (*p* = 0.02, odds ratio (OR) 6.2, 95% confidence interval (CI) 1.3–30.2), “Charlson comorbidity index (CCI) > 2” (*p* = 0.04, OR 4.0, 95% CI 1.0–15.5), “early postoperative complications (PSIs)” (*p* = 0.001, OR 17.1, 95% CI 3.1–96.0) and “PMV > 24 hrs” (*p* = 0.002, OR 13.0, 95% CI 2.7–63.8) as significant and independent predictors for early postoperative mortality. The present study indicates PMV to significantly correlate to elevated early postoperative mortality rates following stabilization surgery for PSD. These results might entail further scientific efforts to investigate PMV as a so far underestimated negative prognostic factor in the surgical treatment of PSD.

**Supplementary Information:**

The online version contains supplementary material available at 10.1007/s10143-023-02016-1.

## Introduction

Pyogenic spondylodiscitis (PSD) represents about 3–5% of all forms of osteomyelitis with a continuously increasing incidence up to currently 6.2–7.4/100,000 patients per year worldwide [1, 2]. The German Federal Statistical Office (2015) reports an age-standardized yearly incidence rate of 30/250,000 [3]. In addition to specific antibiotic treatment regimens, surgical intervention is often recommended and necessary due to the prevention of secondary cumulative effects such as spinal instability and severe neurological morbidity [3, 4]. The aim of spinal surgery is to remediate the foci of infection in the spine and the adjacent tissue, to relieve the pain, and to reconstruct spinal stability [3, 4]. However, surgical treatment of PSD as a systemic and life-threatening infectious disease might be accompanied by the need of postoperative intensive care which might entail prolonged intervals of postoperative mechanical ventilation. There is evidence that prolonged mechanical ventilation (PMV) has an additional critical impact on patient morbidity and mortality which in turn might worsen intended operative benefit [5–11]. The aim of the present study was to analyze the impact of PMV on early postoperative mortality in patients that had undergone stabilization surgery for PSD. In a second step, the authors aimed at identifying preoperatively collectable factors for PMV occurrence in surgical treatment of PSD.

## Methods

### Patients

All consecutive patients with the ICD code diagnosis of discitis or pyogenic vertebral osteomyelitis with infection of the intervertebral disc aged ≥ 18 years who had undergone surgical procedures with instrumentation and/or fusion for PSD at the authors’ neurosurgical department between 2012 and 2018 were entered into a computerized database (SPSS, version 25, IBM Corp., Armonk, NY). Information collected for each patient included sociodemographic characteristics, location of the spinal infectious disease, number of affected spinal levels, and neurological status at admission among others. Indication for an open surgical procedure was given in case of neurological impairment, osseous destruction with spinal instability/deformity or stenosis of spinal canal and compression of neuronal structures, relevant epidural empyema and/or ventral/paravertebral abscess inaccessible for puncture, failure of conservative treatment with progressive disease, or intractable pain.

Depending on the site, extent and severity of the infectious spinal disease dorsolateral as well as ventral approaches were performed. In case of severe instability, a decision for a staged procedure (360° stabilization) was made. Pathogen detection was defined as positive microbiological cultivation out of preoperatively taken blood cultures and/or out of intraoperatively obtained bone or disc samples with pathogen-specific resistograms. In case of unsuccessful germ cultivation, diagnosis of PSD was made by means of histopathological pathogen detection from intraoperative bone and/or disc samples as well as consistent correlation of radiological signs, systemic inflammatory blood values, and intraoperative macroscopic and microscopic tissue appearance. In case of successful germ detection, a specific antimicrobial therapy was started in accordance to pathogen-specific resistograms. In some patients with septic constellations and/or preexisting antibiotic therapy, empirical and broad antibiotic therapy was necessary. Antibiotics were administered intravenously at least in the first 2 weeks with subsequent potential oralization after C-reactive protein (CRP) dropping below 10 mg/L. A minimum of 6 weeks of antibiotic therapy was standard. Selection of the antimicrobial therapy according to the pathogen spectrum and resistogram occurred in close consultation with the local Institute of Microbiology.

PMV was defined as an invasive ventilation period of > 24 h after initial stabilization surgery as previously described [10]. Therefore, the group of PMV patients was made up of patients, who remained mechanically ventilated for > 24 h during the immediate postoperative course, whereas patients with initial postoperative extubation and secondary mechanical ventilation for > 24 h did not rank among this group. The comorbidity burden was objectified using the Charlson comorbidity index (CCI) as previously described [12, 13]. Preoperative systemic inflammatory status was assessed by means of CRP and white blood cell (WBC) levels. Laboratory analysis of CRP and WBC was performed within 12 h of admission as part of routine laboratory testing. Dichotomization of WBC (normal range 3.9–10.2 G/L) and CRP values (normal range 0–3 mg/L) was performed according to previously described cutoffs (WBC ≤ 12 G/L and > 12 G/L; CRP ≤ 10 mg/L and > 10 mg/L) that have been correlated to moderate systemic infection in PSD as well as several other diseases [7–9, 14].

Early postoperative complications were assessed by means of a public available list of events introduced by the Agency for Healthcare Research and Quality and the Center for Medicare and Medicaid Services and referred to as patient safety indicators (PSIs) and hospital-acquired conditions (HACs) [12]. PSIs included the complicative occurrence of acute myocardial infarction, pressure ulcers, iatrogenic pneumothorax, transfusion reactions, perioperative and postoperative hemorrhage, pulmonary embolism, acute postoperative respiratory failure, deep vein thrombosis, wound dehiscence as well as accidental puncture or laceration. Within the group of HACs, screening was performed for pneumonia, catheter-associated urinary tract infection, surgical site infection, postoperative sepsis, blood incompatibility, crushing injury, manifestation of poor glycemic control (diabetic ketoacidosis, non-ketonic hyperosmolar coma, hyperglycemic coma), fall injury, and vascular catheter-associated infection. In addition, to assess complications specific to spinal surgeries, postoperative periods were screened for cerebrospinal fluid (CSF) leakage, postoperative meningitis, implant failure as well as postoperative new or worsened neurological deficits and were classified as spinal surgery-related complications (SSCs). Perioperative complications were defined as any postoperative adverse event with/without further surgical interventions occurring within 30 days after initial surgery.

The study was conducted in accordance with the Declaration of Helsinki and approved by the Ethics Committee of the University Hospital Bonn (approval number 161/20, approval date 20 April 2020). Informed consent was not sought in regard of the retrospective study design.

### Statistical analysis

Data analysis was performed using the computer software package SPSS (IBM Corp. Released 2017, IBM SPSS Statistics for Windows, Version 25.0, Armonk, NY: IBM Corp.) and GraphPad Prism (version 9.2.0 for Windows, GraphPad Software, San Diego, CA, USA, www.graphpad.com). Categorical variables were analyzed in contingency tables using Fisher’s exact test. The Mann-Whitney *U* test was chosen to compare continuous variables as the data were mostly not normally distributed. Results with *p* < 0.05 were considered statistically significant. In addition, in order to determine independent predictors of 30-day mortality in patients with surgically treated PSD, a backward stepwise method was used to construct a multivariable analysis using a binary logistic regression.

## Results

### Patient characteristics

Between January 2012 and December 2018, 177 patients were surgically treated with spinal instrumentation for PSD at our neurosurgical department. Median age at time of surgery was 72 years (IQR 62–78 years) including 68 female (38%) and 109 male patients (62%) (Table 1). The most common location of the infectious disease was the lumbar spine (60%), followed by the thoracic (20%) and the cervical spinal section (13%). One hundred fifty-one of 177 patients (85%) suffered from mono- and bi-level disease; multilevel PSD was present in 26 of 177 patients (15%). Fifty-nine patients (33%) suffered from additional spinal empyema. The most prevalent detected microbiological pathogen was methicillin-susceptible *Staphylococcus aureus* (32%) followed by *Staphylococcus epidermidis* (10%). Clinical admission status revealed PSD-related neurological deficits in 50 of 177 patients (28%). Sixteen patients (9%) died within 30 days following initial stabilization surgery. Twenty-three of 177 patients (13%) received mechanical ventilation of more than 24 h. For further details on patient characteristics, see Table 1.

**Table 1:** Baseline characteristics

		*n* = 177
Median age (IQR) (years)		72 (62–78)
Female sex		68 (38)
Location of disease		
	Cervical	22 (13)
	Thoracic	36 (20)
	Lumbar	107 (60)
	Combined	12 (7)
Level of disease		
	1–2	151 (85)
	≥ 3	26 (15)
Associated spinal empyema		59 (33)
Neurological deficit		50 (28)
Median CCI (IQR)		1 (0–3)
ASA ≥ 3		109 (62)
Median duration of surgery		243 (187–327)
30-Day mortality		16 (9)
Postoperative PMV		23 (13)

*ASA* American Society of Anesthesiology physical status classification system, *CCI* Charlson comorbidity index, *IQR* interquartile range, *n* number of patients, *PMV* prolonged mechanical ventilation

Values represent the number of patients unless indicated otherwise (%)

*Analysis of patient- and disease-related characteristics dependent on the presence of PMV*

**Fig. 1:**
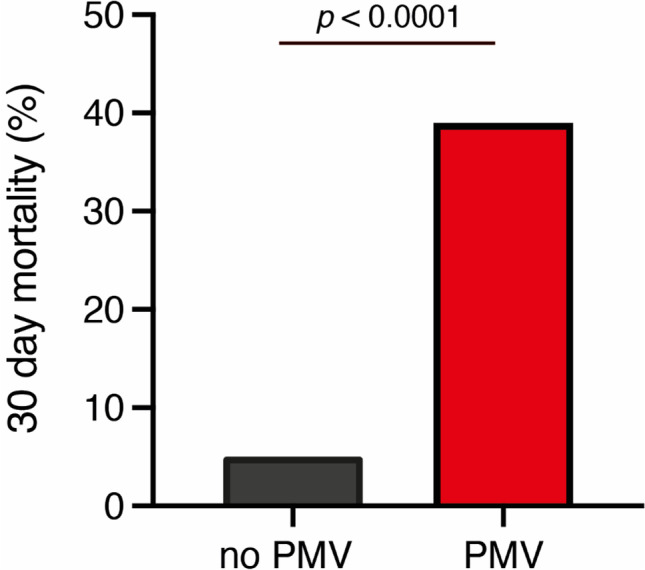
Thirty-day mortality dependent on the occurrence of postoperative PMV. PMV, prolonged mechanical ventilation

**Fig. 2:**
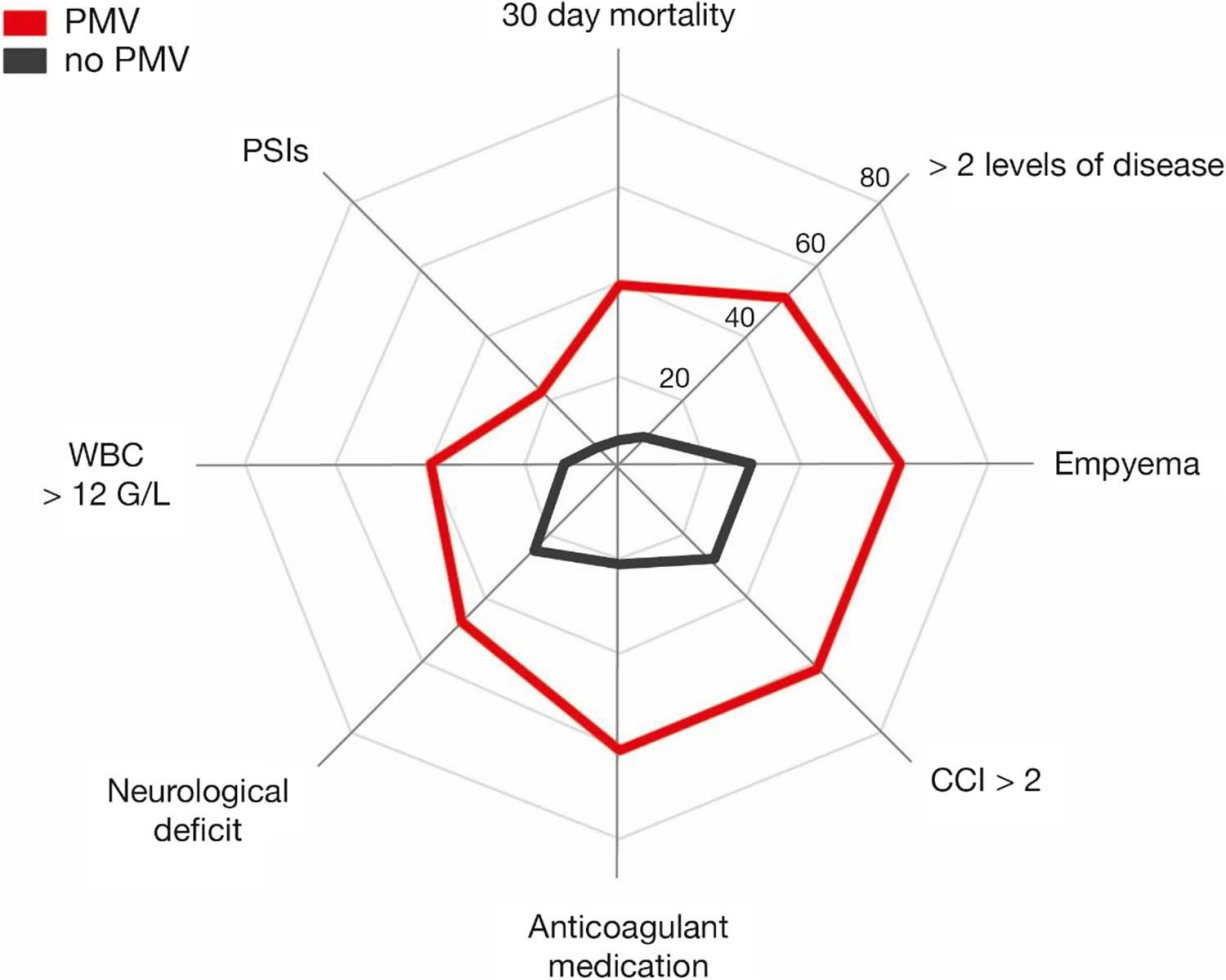
Radar plot depicting patient- and disease-related characteristics dependent on the presence of postoperative PMV in patients with surgically treated pyogenic spondylodiscitis. CCI, Charlson comorbidity index; PMV, prolonged mechanical ventilation; PSIs, patient safety indicators; WBC, white blood cell

Thirty-day mortality in patients with postoperative PMV was 39% (9 of 23 patients) compared to 5% (5 of 154 patients) in patients without postoperative PMV (*p* < 0.0001) (Table 2, Figs. 1 and 2). Patients with cervical PSD or multilevel disease were significantly more likely to have PMV (*p* = 0.01) as shown in Table 2. Similarly, the percentage of patients with PSD-associated spinal empyema, preoperative anticoagulant medication, preoperative neurological deficits, and increased WBC count as an indicator of elevated systemic inflammatory responses was significantly higher in the PMV cohort (Table 2, Fig. 2). Median CCI was significantly higher in the PMV cohort (*p* = 0.004). Nine of 23 patients with PMV (39%) suffered from respiratory functional disorder due to preoperatively diagnosed sepsis from pneumonia, urinary tract infection, and/or spinal source of infection including spinal empyema. Four patients (17%) suffered from exacerbated chronic obstructive pulmonary disease (COPD); 2 of 23 patients (9%) developed perioperative or early massive postoperative pulmonary embolism with the need for further mechanical ventilation. Extubation was unsuccessful due to perioperative acute myocardial infarction in 1 patient (4%). One of 23 patients with PMV (4%) suffered from preoperative hemodynamic instability. Nine of 23 patients with PMV (39%) developed early postoperative complications compared to 24 of 154 patients without PMV (16%) (*p* = 0.02). For detailed information on postoperative complication profiles, see Supplementary Table S1.

**Table 2:** Factors associated with postoperative PMV following stabilization surgery for pyogenic spondylodiscitis

		Patients without PMV *n* = 154	Patients with PMV *n* = 23	*p* value
Median age (years)**		72 (60–78)	73 (68–77)	0.35
Female sex		62 (40)	6 (26)	0.25
Location of disease				0.03
	Cervical	15 (10)	7 (30)	0.01
	Thoracic	31 (20)	5 (22)	0.79
	Lumbar	98 (64)	9 (39)	0.04
	Combined	10 (6)	2 (9)	0.66
Level of disease				< 0.0001
	1–2	140 (91)	11 (48)	
	> 2	14 (9)	12 (52)	
Associated spinal empyema		45 (29)	14 (61)	0.004
Median CCI		1 (0-3)	3 (1-4)	0.004
Preoperative anticoagulant medication		33 (21)	14 (61)	0.004
Preoperative neurological deficit		39 (25)	11 (48)	0.04
Preoperative systemic inflammation levels				
	CRP > 10 mg/L	132 (86)	23 (100)	0.08
	WBC > 12 G/L	17 (11)	9 (40)	0.002
Median time of surgery		243 (185–317)	274 (189–393)	0.27
Early postoperative complications		24 (16)	9 (39)	0.02
	PSIs	9 (6)	5 (22)	0.02
	HACs	10 (6)	4 (17)	0.09
	Specific SSCs	5 (3)	0 (0)	1.0
30-Day mortality		7 (5)	9 (39)	< 0.0001

Values represent the number of patients unless indicated otherwise (%)

*CCI* Charlson comorbidity index, *CRP* C-reactive protein, *HAC* hospital-acquired conditions, *IQR* interquartile range, *PSIs* patient safety indicators, *SSCs* spinal surgery-related complications, *WBC* white blood cells

**Median (IQR)

### Multivariable analysis identifies PMV as an independent predictor of early postoperative mortality

We conducted a multivariable analysis in order to identify independent predictors of 30-day mortality following stabilization surgery in patients with PSD. Under consideration of the variables location of disease, level of disease, associated spinal empyema, median CCI, preoperative anticoagulant medication, preoperative neurological deficit, preoperative WBC > 12 G/L as systemic inflammation level, early postoperative complications as reflected by the rate of PSIs, and PMV > 24 h, the multivariable analysis identified “spinal empyema” (*p* = 0.02, odds ratio (OR) 6.2, 95% confidence interval (CI) 1.3–30.2), “CCI > 2” (*p* = 0.04, OR 4.0, 95% CI 1.0–15.5), “early postoperative complications (PSIs)” (*p* = 0.001, OR 17.1, 95% CI 3.1–96.0) and “PMV > 24 h” (*p* = 0.002, OR 13.0, 95% CI 2.7–63.8) as significant and independent predictors for early postoperative mortality (Nagelkerke’s *R*^2^ 0.5) (for detailed information, see Table 3).

**Table 3:** Multivariable regression analysis for predictors of early postoperative mortality

Factors	Adjusted OR	95% CI	*p* value
Location of disease (cervical)	1.2	0.2–7.5	0.8
Level of disease (> 2)	1.1	0.2–6.2	0.9
Associated spinal empyema	6.2	1.3–30.2	**0.02**
CCI > 2	4.0	1.0–15.5	**0.04**
Preoperative anticoagulant medication	1.3	0.3–5.5	0.7
Preoperative neurological deficit	1.4	0.3–5.8	0.6
Preoperative WBC > 12 G/L	2.0	0.4–10.1	0.4
Early postoperative complications (PSIs)	17.1	3.1–96.0	**0.001**
PMV > 24 h	*13.0*	*2.7–63.8*	***0.002***

*CCI* Charlson comorbidity index, *CI* confidence interval, *OR* odds ratio, *PMV* postoperative prolonged mechanical ventilation, *PSIs* patient safety indicators, *SD* standard deviation, *WBC* white blood cells

### Multivariable analysis identifies preoperatively collectable predictors of PMV occurrence

Having identified PMV > 24 h to significantly result in elevated early postoperative mortality rates following surgery for PSD, a multivariable analysis was performed in order to demask preoperatively collectable predictors for PMV occurrence. Under consideration of the variables age, location of disease, level of disease, associated spinal empyema, median CCI, preoperative anticoagulant medication, preoperative neurological deficit, and preoperative WBC > 12 G/L as systemic inflammation level, the multivariable analysis identified “location of disease (cervical)” (*p* = 0.02, OR 4.6, 95% CI 1.3–16.8), “level of disease (> 2)” (*p* < 0.001, OR 10.0, 95% CI 2.9–43.3), “preoperative anticoagulant medication” (*p* = 0.009, OR 1.3, 95% CI 0.3–5.5), and “preoperative WBC > 12 G/L” (*p* = 0.009, OR 4.6, 95% CI 1.5–14.2) as significant and independent predictors for PMV occurrence (Nagelkerke’s *R*^2^ 0.4) (for detailed information, see Supplementary Table S2).

## Discussion

PSD is a systemic and life-threatening infectious disease [10]. The high average age of mostly multiple affected patients is critical to extensive surgical procedures and therefore often associated with the need for postoperative intensive care treatment [11, 15]. Nowadays, intensive care therapy often includes mechanical ventilation. Ventilation therapy is more often protracted in critically ill and multimorbid patients [16]. PMV has already been revealed to be a negative prognostic factor for survival in other diseases [7, 9–11]. In a French study of 164 patients with traumatic tetraplegia, 87% were mechanically ventilated and the MV duration was found to be an independent factor for poor neurologic outcome [8]. To our knowledge, this is the first study that identifies PMV as an independent factor for early postoperative mortality in patients suffering from pyogenic infection of the spine. The overall 30-day mortality in our population was 9% which is in accordance with the reported high mortality rates in patients with PSD ranging from 4% up to 15% [1, 14, 15, 17–19]. Koslow et al. found a marked increase of mortality up to even 24% in patients with PSD and accompanying endocarditis [20]. In another recent study by Yagdiran et al., the long term mortality rates were found to increase to 23% over a 2-year follow-up [21], but this may be rather explained by the course of age and high comorbidity than the infectious disease at onset. In the PMV group, the 30-day mortality rate of 39% was nearly eightfold higher than in the non-PMV group. Furthermore, we found a significant higher CCI value, higher preoperative systemic inflammation levels, and a higher rate of associated spinal empyema. This indicates a higher comorbidity burden and a higher severity of PSD with a widespread inflammation situation in the PMV group. Our findings are consistent with the literature on a prolonged ventilator weaning following the acute phase of sepsis in patients with spinal cord injury [22]. Also, the significantly higher rate of preoperative oral anticoagulant medication in our population is conclusive to a higher CCI in terms of concomitance of cardiovascular and thromboembolic diseases.

The findings of the present study may raise awareness of a subgroup of patients with PSD—that is the patient cohort exhibiting at least 24 h of mechanical ventilation after spinal instrumentation. This vulnerable subpopulation of patients is at a high risk of early postoperative mortality and might require special attention in preoperative and perioperative patient care. In addition, it would be useful to determine preoperative factors in further studies to identify this highly vulnerable patient group at an early stage in order to improve the treatment goals and to minimize further treatment risks.

### Limitations

The present study has several limitations. Acquisition of data was retrospective which comes with all known and well-described kinds of bias. Patients were not randomized, but treated according to the institutional Standard Operation Procedure for PSD which is in line with the German S2k Guidelines. In addition, the group of 23 patients with PMV is quite small and therefore does not allow for a detailed correlation of ventilation time and early mortality.

The study design did not allow for measurement of long-term outcome, which we know as an important factor for making a treatment decision [23]. Further, according to the “one-in-ten rule” implicating only one predicitve variable to be identified for every ten events [23], the results of the mutlivariable regression analysis with three predictors for 30-day mortality has to be interpreted with constraint. Nevertheless, the present study is the first to investigate PMV as a negativ prognostic parameter in the setting of PSD. The authors intend to use these data as a basis for the initiation of further studies and correlation analysis.

## Conclusions

Postoperative PMV significantly correlates to elevated early postoperative mortality rates following stabilization surgery for PSD. The present study further identifies several preoperatively collectable predictors of PMV occurrence which might help to preoperatively unmask patients who are at a high risk of unfavorable prolonged postoperative intensive care and elevated early postoperative mortality. These results might entail further scientific efforts to comprehensively investigate PMV as a so far underestimated negative prognostic factor in the surgical treatment of PSD.

### Supplementary information

ESM 1 (DOCX 14.2 KB)
