# Supplementary material for: Postoperative prolonged mechanical ventilation in patients with surgically treated pyogenic spondylodiscitis: a surrogate endpoint for early postoperative mortality
Source: Neurosurg Rev. 2023 May 9;46(1):113. doi: 10.1007/s10143-023-02016-1 (PMC10169897; doi:10.1007/s10143-023-02016-1)
Supplement: Supplementary file 1 — ESM 1 (DOCX 14.2 KB) [file 10143_2023_2016_MOESM1_ESM.docx]

**Supplementary Table S1: Overview of early postoperative complication profiles***

| Number of patients with complications | | 33 |
| --- | --- | --- |
| PSIs | | 14 (42) |
|  | Postoperative hemorrhage | 6 (18) |
|  | Postoperative pulmonary embolism | 5 (15) |
|  | Postoperative acute myocardial infarction | 3 (9) |
| HACs | | 14 (42) |
|  | Pneumonia | 1 (3) |
|  | Postoperative sepsis from urinary tract infection | 1 (3) |
|  | Surgical site infection | 9 (27) |
| Specific SSCs | | 5 (15) |
|  | CSF leakage | 2 (6) |
|  | Postoperative meningitis | 1 (3) |
|  | Postoperative new neurological deficit | 1 (3) |
|  | Implant failure | 1 (3) |

*Values represent number of patients unless indicated otherwise (%)

CSF, cerebrospinal fluid; HAC, hospital-acquired conditions; N., number; PSIs, patient safety indicators; SSCs, spinal surgery related complications.

**Supplementary Table S2: Multivariable regression analysis for preoperatively collectable predictors of PMV > 24 hrs**

| **Factors** | **Adjusted OR** | **95% CI** | **p-value** |
| --- | --- | --- | --- |
| Age > 65 yrs | 1.5 | 0.4-6.2 | 0.6 |
| Location of disease (cervical) | 4.6 | 1.3-16.8 | **0.02** |
| Level of disease (> 2) | 10.0 | 2.9-34.3 | **< 0.001** |
| Associated spinal empyema | 1.6 | 0.5-5.2 | 0.5 |
| CCI > 2 | 0.4 | 0.1-1.3 | 0.1 |
| Preoperative anticoagulant medication | 1.3 | 0.3-5.5 | **0.009** |
| Preoperative neurological deficit | 1.4 | 0.3-5.8 | 0.6 |
| Preoperative WBC > 12 G/L | 4.6 | 1.5-14.2 | **0.009** |

CCI, Charlson Comorbidity Index; CI, confidence interval; hrs, hours; OR, Odds ratio; PMV, postoperative prolonged mechanical ventilation; SD, standard deviation; WBC, white blood cells; yrs, years.
